# DNA-induced liquid phase condensation of cGAS activates innate immune signaling

**DOI:** 10.1126/science.aat1022

**Published:** 2018-07-05

**Authors:** Mingjian Du, Zhijian J. Chen

**Affiliations:** 1 Department of Molecular Biology, University of Texas Southwestern Medical Center, Dallas, TX 75390-9148; 2 Center for Inflammation Research, University of Texas Southwestern Medical Center, Dallas, TX 75390-9148; 3 Howard Hughes Medical Institute, University of Texas Southwestern Medical Center, Dallas, TX 75390-9148

## Abstract

The binding of DNA to cyclic GMP-AMP synthase (cGAS) leads to the production of the secondary messenger cyclic GMP-AMP (cGAMP), which activates innate immune responses. Here, we show that DNA binding to cGAS robustly induced the formation of liquid-like droplets in which cGAS was activated. The disordered and positively charged cGAS N-terminus enhanced cGAS–DNA phase separation by increasing the valencies of DNA binding. Long DNA was more efficient in promoting cGAS liquid phase separation and cGAS enzyme activity than short DNA. Moreover, free zinc ion enhanced cGAS enzyme activity both in vitro and in cells by promoting cGAS–DNA phase separation. These results demonstrated that the DNA-induced phase transition of cGAS promotes cGAMP production and innate immune signaling.

## One Sentence Summary

The DNA-sensing enzyme cGAS forms liquid droplets to stimulate innate immune responses.

**Fig. 1:**
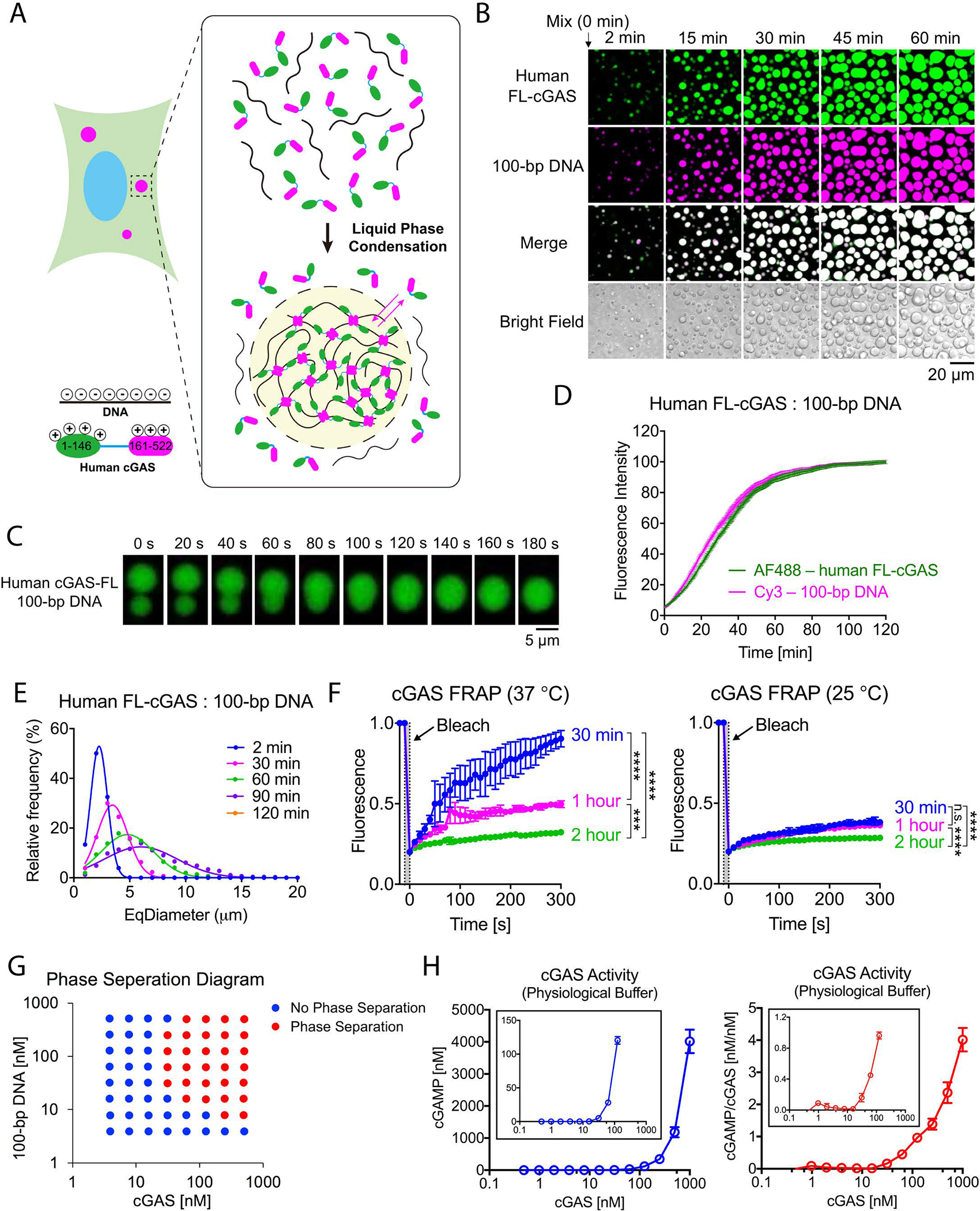
DNA binding to cGAS induces the formation of liquid-like droplets in which cGAS is activated. (A) Schematic of hypothetical cGAS–DNA interactions that drive liquid phase condensation. (B) Time-lapse imaging of cGAS–DNA phase separation. Liquid droplets formed after mixing 10 μM full-length human cGAS (3% Alexa 488-labeled) with 10 μM 100-bp DNA (2% Cy3-labeled) and matured over 60 min. The images shown are representative of all fields in the well. (C) Time-lapse micrographs of merging droplets that formed as described in (B). Data are representative of at least ten merging droplets. (D) Fluorescence intensities of cGAS–DNA liquid droplets forming over the time course of 120 min. Data were normalized to 100% by maximal fluorescence intensity. Values shown are means ± SD. N = 4 images. AF488: Alexa Fluor 488. (E) EqDiameter frequency distribution of cGAS–DNA liquid droplets formed at the indicated time points. EqDiameter: the diameter of a circle with the same area as the measured object. cGAS: 5 μM; DNA: 5 μM. (F) Fluorescence recovery after photobleaching (FRAP) of cGAS–DNA liquid droplets. Bleaching was performed at the indicated time points after cGAS (10 μM) and DNA (10 μM) were mixed, and the recovery was allowed to occur at 25 °C (left) or 37 °C (right). Time 0 indicates the start of recovery after photobleaching. Shown are the mean ± SD. N = 3 liquid droplets. One-way ANOVA; p-value: > 0.0332 (n.s.), 0.0332 (*), 0.0021 (**), 0.0002 (***), < 0.0001 (****). (G) Phase separation diagram of full-length human cGAS and 100-bp DNA at indicated concentrations. Blue dots: no phase separation; red dots: phase separation. (H) Left panel: cGAMP production by indicated concentrations of cGAS in the presence of ATP, GTP, and HT-DNA. cGAMP production at low cGAS concentrations is shown in the inset; right panel: normalized cGAMP production divided by cGAS concentrations. Shown are the mean ± SD. N = 3 assays. Data are representative of at least three independent experiments unless indicated otherwise.

Cyclic GMP-AMP synthase (cGAS) is a DNA sensing enzyme that catalyzes the conversion of GTP and ATP to cGAMP, which activates the adaptor protein STING (1, 2). This in turn induces type-I interferons and other cytokines (3–5). DNA arising in the cytoplasm activates cGAS and drives the formation of cytoplasmic foci containing cGAS and DNA (1). However, the molecular mechanism and functional effect of such cGAS foci are poorly understood. cGAS contains a disordered and positively charged N-terminus and a structured C-terminus harboring a nucleotidyltransferase domain (Core-cGAS). Both the N- and C-termini of cGAS bind to DNA irrespectively of DNA sequence (6–11). We hypothesized that such multivalent interactions could lead to the formation of large membrane-less protein foci through liquid phase separation (Fig. 1A). This physicochemical process has emerged as a key mechanism underlying the formation of cellular bodies such as P granules and nucleoli (12–20).

To test if DNA binding induces phase separation of cGAS in vitro, we incubated fluorescently labeled cGAS protein (Fig. S1A) with double-stranded DNA oligos (100 bp; Table S1; see Methods). Upon mixing, cGAS and DNA formed micrometer-sized liquid droplets within 2 min (Fig. 1B and Movie S1). Small liquid droplets fused into larger ones (Fig. 1C), accompanied by increased fluorescence intensity and larger equivalent diameter (EqDiameter; Fig. 1D, 1E and Movie S1). Fluorescence recovery after photobleaching (FRAP) experiments showed that when bleaching was performed 30 minutes after the initiation of phase separation, the fluorescence of cGAS or DNA was efficiently recovered in a temperature-dependent manner (Fig. 1F and S1B–D). In contrast, when bleaching occurred 2 hours after mixing cGAS and DNA, the fluorescence recovery was much slower (Fig. 1F; Fig. S1D). Thus, cGAS and DNA molecules within the liquid droplets are mobile and exhibit dynamic internal rearrangement in the early phase, but gradually undergo a liquid-to-solid transition and mature into a gel-like state (Fig. 1F) (19, 21).

cGAS and DNA formed liquid droplets when the concentration of each was above 30 nM (Fig. 1G) in a buffer mimicking physiological ion concentrations and composition of the cytoplasm (physiological buffer; see Methods). Measurements of cGAMP production by cGAS revealed that the specific activity of cGAS significantly increased at concentrations above 30 nM (Fig. 1H; see Methods). The in vitro cGAS–DNA phase transition was weakened by increasing salt (NaCl) concentrations (Fig. S2A), suggesting that ionic interactions between cGAS and DNA were important for the phase transition. A 45-bp dsDNA commonly used for the stimulation of the cGAS pathway (immune stimulatory DNA or ISD) also robustly induced cGAS liquid droplet formation (Fig. S2B–D). This effect was abolished by treatment with Benzonase, which degrades DNA (Fig. S2E). cGAS also formed liquid droplets with 45-bp dsRNA (Fig. S2F), but RNA did not activate cGAS to produce cGAMP (Fig. S2G). These results are consistent with previous structural studies indicating that DNA but not RNA binding induces a conformational change that activates cGAS (7). Thus, liquid phase separation is not sufficient for cGAS activation in the absence of the correct conformational change induced by DNA.

The cGAS–DNA phase separation was unaffected by adding ATP, GTP, or a combination of both (Fig. S3A). Moreover, ATP or GTP could be partitioned into and enriched in the cGAS–DNA liquid droplets (Fig. S3B–C). FRAP experiments showed that ATP rapidly exchanged into and out of the cGAS–DNA droplets (Fig. S3D).

**Fig. 2:**
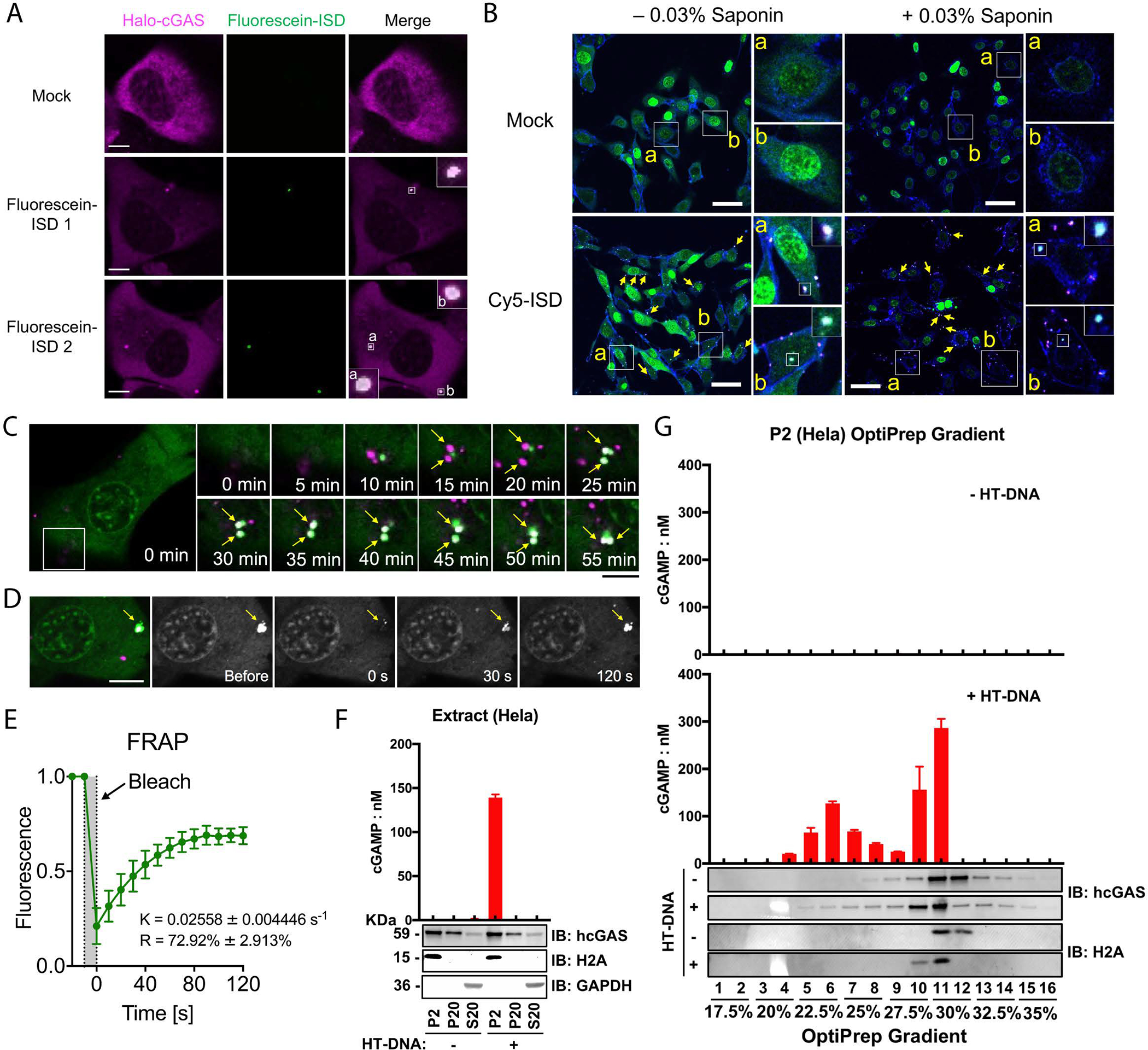
DNA-induced liquid phase separation of cGAS in cells. (A) Representative live cell images of cGAS–DNA puncta formation after transfection of fluorescein-ISD into BJ cells stably expressing Halo-cGAS, which was covalently labeled with TMR. Zoomed images indicate cGAS–DNA puncta (Boxes). Scale bar: 10 μm. These images are representative of at least 10 cells. (B) MEF cells stably expressing GFP–cGAS were transfected with Cy5-ISD for 4 hours, followed by permeabilization of the cells with saponin and fluorescence microscopy. Shown in blue is the plasma membrane marker wheat germ agglutinin. Scale bar: 50 μm. These images are representative of at least 5 fields examined. (C) Time-lapse micrographs of cGAS (green) and DNA (magenta) puncta formation and fusion (time 0 represents 30 min after Cy5-ISD45 transfection). Scale bar: 15 μm. The fusion events existed in all 8 fields examined. (D) Representative micrographs of cGAS–DNA puncta before and after photobleaching (arrow, bleach site). Scale bar: 15 μm. These images are representative of at least 3 cells in which the cGAS–DNA puncta were photobleached. (E) Quantification of cGAS–DNA puncta FRAP over a 120-second time course. K: exponential constant. R: normalized plateau after fluorescence recovery. Shown are means ± SD. N = 3 cGAS–DNA puncta. (F) Subcellular fractionation of cGAS activity in DNA-transfected cells. HeLa cells transfected with or without HT-DNA were fractionated by differential centrifugation as depicted in Fig. S4B. Fractions were incubated with ATP and GTP followed by measurement of cGAMP. Fractions were also analyzed by immunoblotting with antibodies against histone H2A (nuclear marker), GAPDH (cytoplasmic marker), or cGAS. (G) The P2 fractions from (F) were further separated by Optiprep gradient ultracentrifugation and cGAS activity in different fractions were measured as in (F). Fractions from cells not transfected with DNA had no cGAS activity (upper panel). Error bars in (F) and (G) represent the variation range of duplicate assays. Data are representative of at least three independent experiments.

We next sought to examine the cGAS–DNA foci formation in cells. In the human fibroblast cell line BJ-5ta stably expressing a Halo-tagged cGAS, cGAS formed puncta with fluorescein-labeled ISD in the cytoplasm (Fig. 2A and S4A). To confirm that cGAS formed large granules with DNA in the cytoplasm, we used cGAS-deficient MEF cells reconstituted with GFP-cGAS, transfected the cells with Cy5-ISD and then permeabilized the cells with saponin (22). cGAS formed puncta with ISD and remained in the cytoplasm after saponin treatment (Fig. 2B). The cGAS–DNA foci exhibited liquid-like properties as evidenced by that two foci could fuse with each other (Fig. 2C). Furthermore, upon photobleaching, cGAS in the foci exhibited near-complete fluorescence recovery within 120 seconds (Fig. 2D, 2E), indicating that cGAS exhibits a dynamic liquid-like behavior within cellular granules.

To determine the functional consequence of cGAS liquid droplet formation, we transfected Hela cells with herring testis DNA (HT-DNA), and measured cGAS activity in subcellular fractions (Fig. S4B). Most cGAS activity was present in the pellet after centrifugation at 2,000 × *g* (P2), which mainly contains the nuclei and heavy particles (Fig. 2F). Further separating the P2 fraction by iodixanol (OptiPrep) density gradient ultracentrifugation revealed two distinct pools of cGAS activity. The first pool was in very heavy fractions (20%–25% iodixanol; Fig. 2G), which were separated from the nuclei (27.5%–30% iodixanol). These results suggest that cGAS formed heavy particles with transfected DNA that were distinct from cellular organelles and vesicles, and these particles contained active cGAS. The second pool of cGAS activity was in fractions that contained the nuclei. However, it remains to be determined whether this activity came from cGAS within the nuclei or some cGAS particles that co-sedimented with the nuclei. Similar results were also obtained in human monocytic THP-1 cells (Fig. S4C, S4D).

**Fig. 3:**
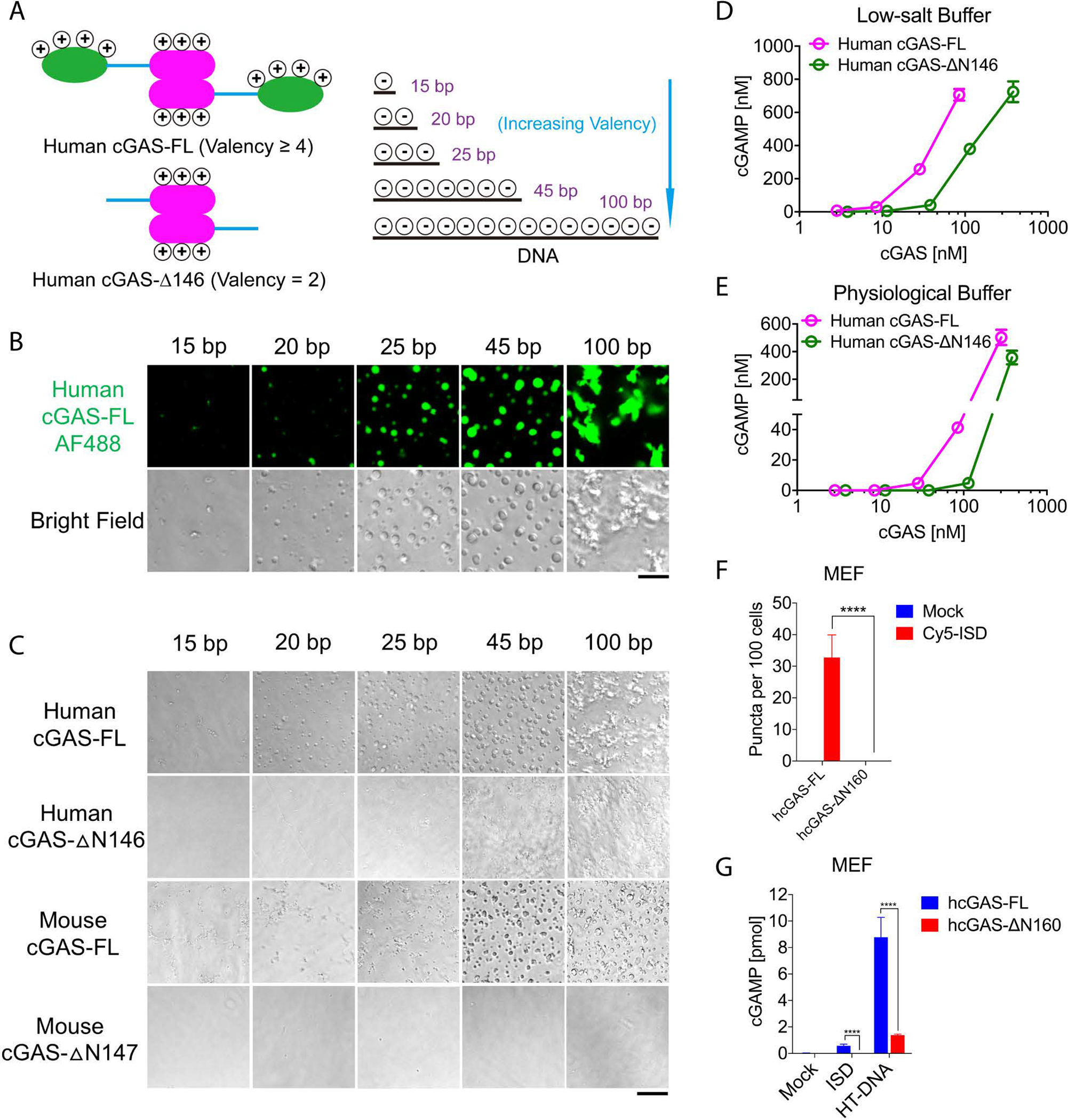
Multivalent interactions drive cGAS–DNA condensation and promote cGAS activation. (A) Schematic of hypothetical cGAS and DNA valencies. (B) Representative images of phase separation by mixing cGAS (10 μM) with dsDNA of different lengths (10 μM) in physiological buffer. Scale bar: 10 μm. AF488: Alexa Fluor 488. (C) Bright-field images of phase separation by mixing DNA of different lengths with full-length or N-terminally truncated human or mouse cGAS as indicated. Scale bar: 20 μm. The images shown in (B) and (C) are representative of all fields in the wells. (D & E) cGAMP production by different concentrations of recombinant full length or ΔN human cGAS in low-salt buffer (D) or physiological buffer (E). Shown are the mean ± SD. N = 3 assays. (F) Quantification of cGAS–DNA puncta by imaging of MEF cells expressing GFP-tagged full length human cGAS or ΔN160-cGAS after transfection of Cy5-ISD. Representative images are shown in Figure S6. Values shown are means ± SD. N = 5 images. (G) cGAMP production in the MEF cells expressing full length or ΔN160 human cGAS after transfection with ISD or HT-DNA. Values are means ± SD. N = 3. Multiple t-tests; p-values (for F and G): > 0.0332 (n.s.), 0.0332 (*), 0.0021 (**), 0.0002 (***), < 0.0001 (****). cGAS expression levels are shown in Figure S5F. Data are representative of at least three independent experiments.

Multivalent interactions drive liquid phase separation (16). Long DNA has more binding sites (valency) for cGAS than short DNA, and full-length cGAS has higher valency for DNA than core-cGAS (Fig. 3A) (6, 7, 11). To test whether cGAS–DNA liquid phase separation is driven by the valency of cGAS and DNA interactions, we incubated purified full-length (FL-cGAS) and N-terminally truncated (ΔN146) cGAS with DNA of different lengths in the physiological buffer (15 mM NaCl, 135 mM KCl) or a buffer containing 300 mM NaCl. Human FL-cGAS formed more numerous and larger liquid droplets with longer DNA (Fig. 3B). Both human and mouse FL-cGAS exhibited stronger phase separation than N-terminally truncated cGAS with DNA of the same length either in the physiological buffer (Fig. 3C) or at 300 mM NaCl (Fig. S5C). The enzymatic activity of FL-cGAS in the presence of HT-DNA was stronger than that of ΔN146-cGAS in both low-salt buffer (Fig. 3D) and physiological buffer (Fig. 3E).

To investigate the role of the cGAS N-terminus in cells, we reconstituted cGAS-deficient MEF cells with human FL-cGAS or ΔN160-cGAS (Fig. S5F). Following transfection with Cy5-ISD, FL-cGAS formed puncta with Cy5-ISD in the MEF cells, whereas ΔN160-cGAS formed fewer puncta (Fig. 3F, S6). cGAMP production was also higher in cells stably expressing FL-cGAS than those expressing ΔN160-cGAS upon transfection with 45-bp ISD or HT-DNA (Fig. 3G; cGAS expression levels are shown in Fig. S5F).

**Fig. 4.**
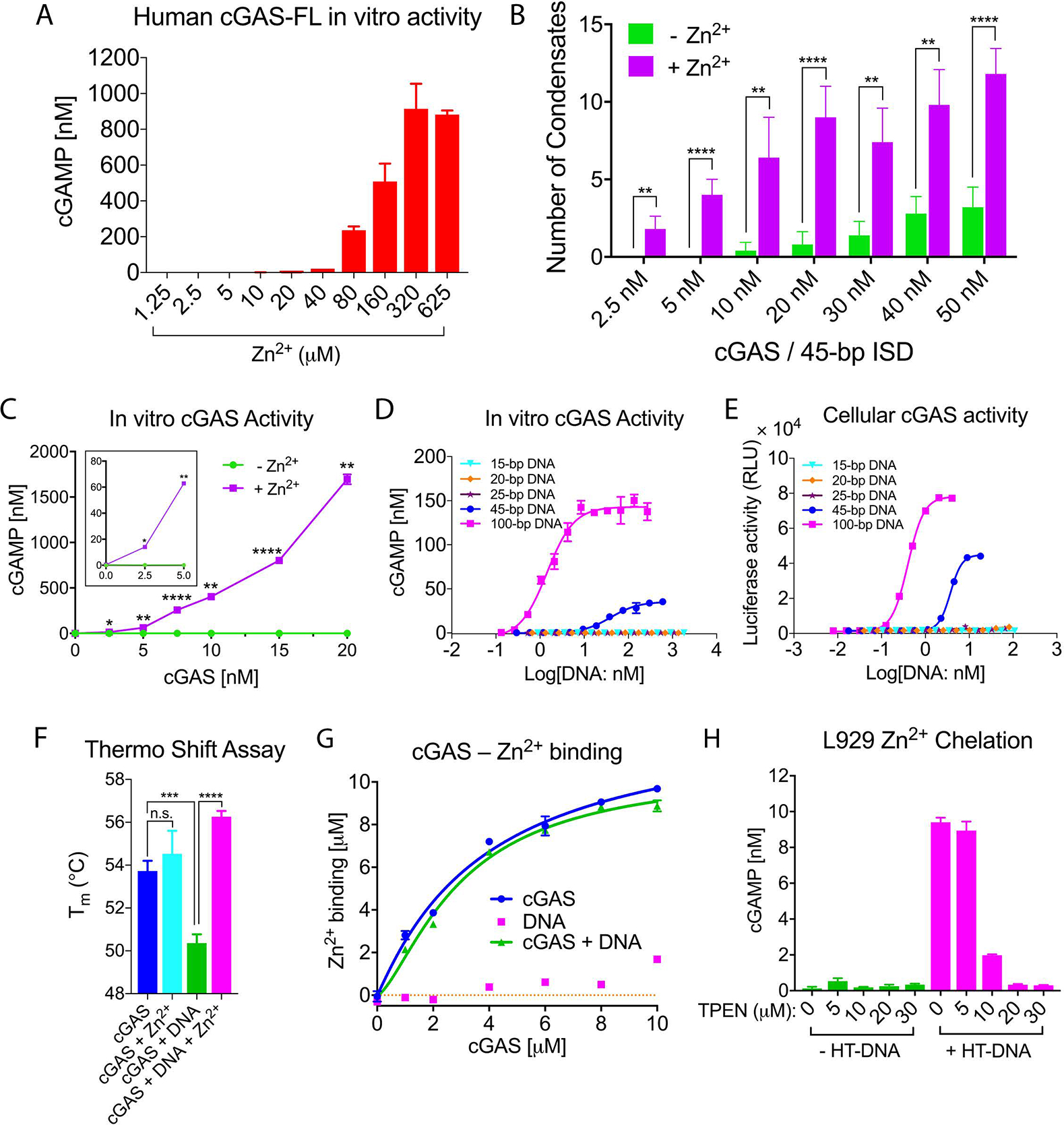
Zinc ion promotes DNA-induced phase separation and activation of cGAS. (A) Zn^2+^ enhances cGAS activation in vitro. Recombinant human full-length cGAS (15 nM) was incubated with ATP, GTP, and DNA in a physiological buffer containing indicated concentrations of Zn^2+^, followed by measurement of cGAMP production. (B) Quantification of cGAS–DNA condensates in the presence or absence of zinc. Liquid-phase condensates formed after mixing Alexa Fluor 488-labeled full-length human cGAS with 45-bp Cy3-labeled ISD at indicated concentrations of each in physiological buffer with or without Zn^2+^ (200 μM). Images were then captured by confocal microscopy and representative images are shown in Figure S7D. Values are means ± SD. N = 5 images. Multiple t tests. (C) cGAMP production in physiological buffer containing HT-DNA and different concentrations of cGAS in the presence or absence of Zn^2+^ (200 μM). The activity of cGAS at low concentrations is shown in the inset. Multiple t tests. (D) cGAMP production by 10 nM cGAS in physiological buffer containing 200 μM Zn^2+^ and different concentrations of DNA of indicated lengths. (E) THP1-Lucia ISG cells, which harbor a luciferase gene under the ISG54 promoter, were transfected with indicated DNA for 24 hours followed by measurement of the secreted luciferase activity. RLU: relative luciferase unit. (F) Thermo shift assay to measure the stability of cGAS or cGAS–DNA complex in the presence or absence of Zn^2+^ (200 μM). Tm: protein melting temperature. Values are means ± SD. N = 3. Unpaired t test. (G) Measurement of cGAS binding to zinc. Zinc ion (10 μM) was incubated with various concentrations of DNA, cGAS, or both, and the solution was passed through a centrifugal filter, followed by measuring zinc ion concentration in the filtrate. The K_d_ values of zinc binding to cGAS and cGAS–DNA complex were 3.9 ± 1.3 μM and 3.0 ± 0.4 μM, respectively. (H) Depletion of intracellular zinc inhibits cGAS activation by DNA. L929 cells were incubated with the indicated concentrations of Zn^2+^ chelator TPEN for 2 hours before transfection with HT-DNA. cGAMP production was measured by a bioassay. Images of intracellular zinc depletion are shown in Figure S8B p values for B, C and F: > 0.0332 (n.s.), 0.0332 (*), 0.0021 (**), 0.0002 (***), < 0.0001 (****). Error bars represent the variation range of duplicate assays unless indicated otherwise. Data are representative of at least three independent experiments.

cGAS enzyme activity was much weaker when the assay was carried out in the physiological buffer than in the low-salt buffer (Fig. S5D, S5E). This raises the question of how cGAS is activated in cells. We found that zinc ions significantly promoted the activity of recombinant cGAS in the physiological buffer and this enhancement could be partially replaced by other ions, such as Mn^2+^ or Co^2+^ (Fig. 4A, S7A), which is a general characteristic of enzymes that require zinc (23). Similarly, Zn^2+^ was the most efficient in activating mouse cGAS (Fig. S7B, S7C). The optimal concentrations of Zn^2+^ were 160–625 μM (Fig. 4A, S7C), which is within the physiological concentration range of zinc ions in cells (24, 25). Zn^2+^ at ~200 μM dramatically facilitated DNA-induced cGAS phase separation at low concentrations of cGAS and DNA (Fig. 4B, S7D). Moreover, cGAS activity at low concentrations was dramatically enhanced in the presence of Zn^2+^ (Fig. 4C). At a concentration as low as 2.5 nM, which is below the concentration of cGAS in the cytoplasm of HeLa cells (approximately 8–12 nM; see Methods), cGAS underwent phase transition and catalyzed cGAMP synthesis (Fig. 4B, 4C). At 10 nM cGAS, 100-bp DNA activated cGAS with an EC50 of ~1.4 nM, whereas 45-bp DNA had much weaker activity. DNA at 25 bp or shorter had no detectable activity in the in vitro assay (Fig. 4D). This DNA-length dependent activation of cGAS was largely mirrored in the cellular assay in which DNA was transfected into a THP1 reporter cell line (Fig. 4E).

Using a thermos shift assay (TSA), we found that DNA binding destabilized cGAS but Zn^2+^ stabilized the cGAS–DNA complex (Fig. 4F, S8A). Measurements of free Zn^2+^ concentrations revealed that Zn^2+^ bound to cGAS, but not DNA (Fig. 4G). To determine if zinc plays a role in cGAS activation within cells, we depleted zinc in L929 cells with the zinc-specific chelator N,N,N′,N′-tetrakis(2-pyridinylmethyl)-1,2-ethanediamine (TPEN). Cellular cGAMP production upon HT-DNA transfection was decreased in a TPEN concentration-dependent manner (Fig. 4H, S8B). Under these conditions, TPEN did not affect the viability of L929 cells (Fig. S8C). Live-cell imaging using a zinc-specific fluorescent probe revealed that cGAS–DNA puncta contained zinc (Fig. S8D, S8E). These results showed that zinc facilitated cGAS activation in cells by promoting cGAS phase transition in the presence of cytosolic DNA.

Thus, DNA binding to cGAS induces a robust phase transition to liquid-like droplets, which function as micro reactors in which the enzyme and reactants are concentrated to greatly enhance the production of cGAMP. This mechanism allows cGAS to detect the presence of DNA in the cytoplasm above a certain threshold to trigger a switch-like response. Such a switch-like response is made possible by the multivalent interactions between the DNA-binding domains of cGAS and DNA in a manner that depends on the DNA length. This also provides an explanation for why long DNA activates cGAS more efficiently. The binding between cGAS and DNA involves extensive ionic interactions between the positively charged surfaces of cGAS and negatively charged DNA. Such interactions are vulnerable to cytoplasmic salt concentrations, which may be a mechanism to prevent spurious activation of cGAS by self-DNA below a certain threshold. However, we found zinc ions could significantly enhance cGAS phase separation and its enzymatic activation at physiological salt concentrations. Free zinc ions are mainly stored in organelles such as the mitochondria and the ER (26), and their delivery to the cytosol may be another avenue by which cGAS activity is regulated in cells. DNA binding to cGAS induced formation of cGAS–DNA condensates, which were observed as cytoplasmic foci within cells. Further characterization of the dynamics and composition of the cGAS condensates should provide deeper insights into the mechanism by which cGAS activity is tightly regulated to trigger an appropriate immune response to pathogens while simultaneously avoiding autoimmune reactions to self-tissues.

## Supplementary Material

Supplementary Material

Movie S1
