# Supplementary material for: DNA-induced liquid phase condensation of cGAS activates innate immune signaling
Source: Science. Author manuscript; Available in PMC 2022 Aug 27. (PMC9417938; doi:10.1126/science.aat1022)
Supplement: Supplementary Material [file NIHMS1829168-supplement-Supplementary_Material.pdf]

Science
AAAS

# Supplementary Materials for

## DNA-induced liquid phase condensation of cGAS activates innate immune signaling

Mingjian Du and Zhijian J. Chen*

*Correspondence to: zhijian.chen@utsouthwestern.edu

**This PDF file includes:**

Materials and Methods
Figs. S1 to S8
Table S1
Captions for Movie S1

**Other Supplementary Materials for this manuscript include the following:**

Movie S1

## Materials and Methods

### Reagents and Cell Lines

Cell lines

Hela, BJ-5ta, and L929 cells were from ATCC and grown in DMEM supplemented with 10% fetal bovine serum (FBS) at 37 °C in 5% $CO_2$. THP1 (ATCC) and THP1-Lucia ISG (Invivogen) cells were cultured in RPMI supplemented with 10% FBS at 37 °C in 5% $CO_2$.

To establish the BJ-5ta-Halo-cGAS cell line, BJ-5ta cells were infected with lentiviruses carrying Halo-hcGAS-Flag and a neomycin-resistance gene. After neomycin (400 µg/ml) selection, surviving cells were frozen or passaged for studies.

To establish $MEF^{cGAS\ KO}$-GFP-cGAS and $MEF^{cGAS\ KO}$-GFP-ΔN160cGAS cell lines, primary $MEF^{cGAS\ KO}$ cells were isolated from C57BL/6 $cGas^{-/-}$ mice and immortalized by SV40 T antigen transformation. Immortalized $MEF^{cGAS\ KO}$ cells were infected with lentiviruses carrying GFP-cGAS or GFP-ΔN160cGAS and a puromycin-resistance gene. After puromycin (1 µg/ml) selection, surviving cells were frozen or passaged for studies.

DNA

Herring testis DNA (HT-DNA), DNA oligonucleotides and fluorescently labeled DNA oligonucleotides were from Sigma-Aldrich (Table S1). Double stranded DNA oligonucleotides were generated by annealing sense and anti-sense ssDNA oligos in annealing buffer (20 mM Tris·HCl pH 7.5, 50 mM NaCl) ramping down from 95 °C to 25 °C at 1 °C/min. The annealing efficiencies were examined by HPLC. For dsDNA equal to or longer than 45 bp, the annealing efficiencies were over 99%. For dsDNA shorter than 45 bp, the annealed dsDNA oligos were diluted into a buffer containing 0.1M triethylamine acetate (TEAA), pH 7.0 and 5% acetonitrile and then subjected to HPLC purification by Waters Xbridge OST C18 column (130 Å, 2.5 $\mu$m, 4.6 mm × 50 mm, cat. 186003953) using Dionex UltiMate 3000 HPLC system. An acetonitrile gradient of 5%–12.5% in 0.1 M TEAA pH 7.0 was used to elute the column at a flow rate of 1 ml/min over the course of 50 min, and 1-ml fractions were collected. Appropriate fractions were collected and concentrated by Labconco CentriVap SpeedVac Concentrator, followed by ethanol precipitation. HPLC-purified dsDNA oligos were resuspended into a desired buffer for experimental use (*27*).

Antibodies

Rabbit monoclonal antibodies against human cGAS (Cat.# D1D3G), mouse cGAS (Cat.# D3O8O), human H2A (Cat.# 2578) and human GAPDH (D16H11) were obtained from Cell Signaling Technology. Mouse monoclonal antibodies against GFP and HA were obtained from BioLegend (Cat.# 902602 and 901501 respectively). Mouse monoclonal anti-Flag M2 antibody was from Sigma-Aldrich (Cat.# F1804).

**Expression, Purification and Labeling of Recombinant cGAS**

Full-length human cGAS (hcGAS-FL), ΔN146 human cGAS (hcGAS-ΔN146), full-length mouse cGAS (mcGAS-FL), and ΔN147 mouse cGAS (mcGAS-ΔN147) were expressed and purified from *Escherichia coli*. *E.coli* strain BL21/pLys harboring a $His_6$-SUMO tagged and codon-optimized plasmid encoding each of the cGAS proteins was induced with 0.8 mM IPTG at 18 °C for 20 hours. Bacteria were collected by centrifugation and lysed by sonication in 20 mM Tris-HCl, pH 8.0, 300 mM NaCl, 20 mM imidazole, 5 mM β-mercaptoethanol, and 0.2 mM PMSF. After centrifugation, clear lysate was incubated with Ni-NTA beads (Qiagen), washed with lysis buffer and eluted with 20 mM Tris-HCl, pH 7.5, 150 mM NaCl, 300 mM Imidazole. The $His_6$-SUMO tag was cleaved by SUMO protease (Ulp1) at 4 °C overnight. Cleaved protein was applied to a 1-ml HiTrap Heparin column (GE Healthcare). After washing with 20 mM Tris-HCl, pH 7.5, 500 mM NaCl, cGAS protein was eluted with a gradient of 0.5–1 M NaCl in 20 mM Tris-HCl, pH 7.5. Eluted cGAS protein was subjected to size exclusion chromatography using a Superdex 200 column (GE Healthcare) in 20 mM Tris-HCl, pH 7.5, 150 mM NaCl and the fractions were collected, concentrated, and dialysed against a buffer containing 20 mM Tris-HCl, pH 7.5 and 150 mM NaCl. Recombinant cGAS protein was labeled with Alexa Fluor 488 by using Alexa Fluor™ 488 Protein Labeling Kit (ThermoFisher) and the estimated degree of labeling was 2 moles of Alexa Fluor 488 per mole of recombinant cGAS.

**In vitro Phase Separation Assay**

In general, recombinant cGAS protein (3% Alexa Fluor 488-labeled) was mixed with DNA of a defined length (2% Cy3-labeled) in 96-well plates (Corning) coated with 20 mg/ml BSA (Sigma). Mixtures were incubated and images were captured at indicated times. Phase separation

of recombinant cGAS with 45-bp ISD was performed in 20 mM Tris-HCl, pH 7.5, 150 mM NaCl and 1 mg/ml BSA. Phase separation of recombinant cGAS with 100-bp DNA was performed in 20 mM Tris-HCl, pH 7.5, 300 mM NaCl, 1 mg/ml BSA. Phase diagrams were generated by mixing recombinant cGAS with 45-bp DNA or 100-bp DNA in 20 mM Tris-HCl, pH 7.5, 150 mM NaCl and 1 mg/ml BSA. Phase separation with 45-bp dsRNA was performed in physiological buffer (20 mM Tris-HCl, pH 7.5, 15 mM NaCl, 135 mM KCl, 5 mM Phosphate, 1.5 mM $MgCl_2$, and 1 mg/ml BSA). Phase separation in the presence or absence of zinc (200 μM $ZnCl_2$) was performed by mixing Alexa Fluor 488-labeled cGAS with Cy3-labeled DNA in physiological buffer.

**Image Acquisition and Analysis**

Phase separated droplets were imaged by using Nikon A1R+ confocal microscope with 40× oil objective, Nikon A1 camera, and X-Cite 120LED laser. Imaging power was 0.5% and images were analyzed using ImageJ (Open source Java program from NIH). Time lapse images of cGAS–DNA phase separation were captured using the Time Lapse Acquisition tool of a Nikon A1R+ confocal microscope every 20 seconds over 2 hours. Fluorescence intensities and EqDiameter (the diameter of a circle with the same area as the measured object) of phase separated droplets were quantified using Nikon NIS-Elements AR (Advanced Research) software. The distribution of droplets EqDiameter was plotted using GraphPad Prism 7.

**In vitro FRAP Assays**

Fluorescence recovery after photobleaching (FRAP) experiments were performed on a Nikon A1R+ confocal microscope at either 25 °C or 37 °C. For FRAP of cGAS or DNA, spots of ~2-μm diameter in ~ 10-μm droplets were photobleached with 20% laser power for 1 second using 488-nm and 561-nm lasers. Time lapse images were acquired over a 20-min time course after bleaching with 10 s interval. For FRAP of ATP, droplets with diameter in ~ 5-μm was fully photobleached with 100% laser power for 10 seconds using 640-nm laser. Time lapse images were acquired over a 1-min time course after bleaching with 1.2 s interval. Images were processed by ImageJ. Fluorescence intensities of regions of interest (ROIs) were corrected by unbleached control regions and then normalized to pre-bleached intensities of the ROIs. The corrected and normalized data were fit to the single exponential model by GraphPad Prism 7:

$$I_t = I_0 + (I_\infty - I_0)(1 - e^{-kt})$$

where $I_0$ was the intensity at the start of recovery after bleaching, $I_\infty$ was the plateau intensity, and $k$ was the exponential constant. $\tau$ was calculated by the reciprocal of $k$. The recovery rate was calculated by $I_\infty$ divided by the fluorescence intensity before bleaching. $t_{1/2}$ represents the time point achieving half recovery intensity ($\frac{I_\infty - I_0}{2}$).

**Cellular FRAP Assays**

Cellular fluorescence recovery after photobleaching (FRAP) experiments were performed on a Nikon A1R+ confocal microscope at 37 °C in a live-cell-imaging chamber. MEF$^{cGAS\ KO}$-GFP-cGAS cells were grown on chambered cover glass until it reached the desired density, at which time cells were transfected with 45-bp Cy5-labeled ISD for 4 hours using lipofectamine 2000. cGAS–DNA puncta were fully or partially photobleached with 20% laser power for 2 s using a 488-nm laser. Time-lapse images were acquired over a 5-min time course after bleaching with 10-second interval. Images were processed by ImageJ and FRAP data were fit to a single exponential model by GraphPad Prism 7.

**Live Cell Imaging**

<u>Imaging of cGAS–DNA puncta and zinc ions in BJ-5ta-Halo-cGAS cells</u>

Cells were grown on chambered cover glass to a proper density and then incubated with 5 μM cell-permeant HaloTag TMR ligand (Promega) in the culture medium at 37 °C for 15 min. The cells were rinsed three times with PBS and incubated in culture medium at 37 °C for 30 min. After labeling, cells were transfected with fluorescein-labeled DNA using lipofectamine 2000. Live cell images were captured after 2 hours by using Nikon A1R+ confocal microscope with 40× oil objective, Nikon A1 camera, and X-Cite 120LED laser. Images were analyzed by ImageJ. For detection of intracellular zinc, TMR-labeled BJ-5ta-Halo-cGAS cells were transfected with Cy5-ISD for 2 hours and then rinsed twice with 1× Tyrode's solution (139 mM NaCl, 3 mM KCl, 17 mM $NaHCO_3$,12 mM Glucose, 3 mM $CaCl_2$, and 1 mM $MgCl_2$). Cells were incubated with 5 μM Zinpyr-1 (AdipoGen) in 1× Tyrode's solution at room temperature for 10 min, followed by rinsing with the same solution for 10 min. Cell images were captured and analyzed as above.

cGAS–DNA puncta imaging with MEF$^{cGAS\ KO}$-GFP-cGAS cells or MEF$^{cGAS\ KO}$-GFP-ΔN160cGAS cells

MEF$^{cGAS\ KO}$-GFP-cGAS or MEF$^{cGAS\ KO}$-GFP-ΔN160cGAS cells growing on chambered cover glass were transfected with Cy5-labeled ISD with lipofectamine 2000, and confocal microscopy was performed and analyzed as described above. For time-lapse live-cell imaging, images were acquired over a 4-hour time course with 5-min intervals, followed by data processing using ImageJ.

**Saponin Permeabilization Assay**

MEF$^{cGAS\ KO}$-GFP-cGAS cells growing on chambered cover glass were transfected with 45 bp Cy5-ISD by lipofectamine 2000 for 4 hours. After washing twice with PBS, cells were stained with plasma membrane dye (5 μM Wheat Germ Agglutinin, Alexa Fluor 350 Conjugate, ThermoFisher) for 10 min. Then cells were washed twice with PBS and incubated with 0.03% saponin in PBS at room temperature for 3 min. Cells were washed with PBS and fixed with 4% paraformaldehyde (Electron Microscopy Sciences) in PBS for 10 min. After two more washes in PBS, cells were imaged using Nikon A1R+ confocal microscope with 40× oil objective, Nikon A1 camera, and X-Cite 120LED laser. Images were analyzed by ImageJ.

**cGAS Activity Assay and cGAMP Measurement**

In vitro cGAS reaction was performed by mixing recombinant human or mouse cGAS protein with ATP (5 mM), GTP (300 μM) and HT-DNA (15 ng/μl) in either the low-salt buffer (20 mM Tris-HCl pH 7.5, 5 mM $MgCl_2$, and 0.2 mg/ml BSA) or the physiological buffer (20 mM Tris-HCl, pH 7.5, 15 mM NaCl, 135 mM KCl, 5 mM Phosphate, 1.5 mM $MgCl_2$, and 0.2 mg/ml BSA). After incubation at 37 °C for 2 hours, reaction was terminated by heating at 95 °C for 5 min to denature proteins, which were removed by centrifugation at 20,000 × *g* for 5 min. The supernatant was delivered into THP1-Lucia ISG cells ($0.25 \times 10^6$ in a 50-μl reaction) that were permeabilized with perfringolysin O (PFO; 50 ng/ml). The cells were cultured at 37 °C for 16 h, at which time the secreted luciferase activity in the culture media was measured using CLARIOstar (BMG LABTECH). Different concentrations of cGAMP were used to generate the standard curve for estimating cGAMP concentrations in the reactions.

The cGAMP bioassay described above was also used to measure cGAMP levels in DNA-transfected cells. Cells were transfected with ISD or HT-DNA by lipofectamine 2000 in Opti-MEM medium. Four hours after transfection, cells were harvested and lysed in 50 μl of hypotonic buffer (10 mM Tris-HCl, pH7.5, 5 mM KCl, and 3 mM $MgCl_2$) supplemented with protease inhibitor cocktail. Cell lysates were heated at 95 ℃ for 5 min to denature proteins, which were precipitated by centrifugation. The supernatant containing cGAMP was measured by delivering into THP1-Lucia cells as described above.

**Subcellular fractionation and cGAS Activity Assay**

Subcellular fractionation

Hela or THP1 cells were transfected with HT-DNA by lipofectamine 2000 in Opti-MEM medium for 2 hours before cells ($5 \times 10^7$) were lysed by passing through a 30G1 needle five times in 500 μl hypotonic buffer (10 mM Tris-HCl, pH 7.5, 5 mM KCl, and 3 mM $MgCl_2$) supplemented with a protease inhibitor cocktail. The homogenate was centrifuged at 2,000 × *g* for 10 min and the pellet (P2) was washed with hypotonic buffer and resuspended into a desired buffer for analysis or further fractionation by ultracentrifugation. The supernatant (S2) was centrifuged at 20,000 × *g* for 10 min and the pellet (P20) was washed with hypotonic buffer and resuspended into a desired buffer for analysis. The supernatant was collected as S20 for analysis.

P2 fractionation by Optiprep gradient and cGAS activity assay

P2 pellet was resuspended in 200 μl of 17.5 % Optiprep with Iso-osmotic Buffer (20 mM Tris-HCl, pH 7.5, 250 mM Sucrose, 25 mM KCl, and 5 mM $MgCl_2$). Optiprep solutions at different densities (20%, 22.5%, 25%, 27.5%, 30%, 32.5%, and 35% in Iso-osmotic buffer) were prepared and used to generate a discontinuous gradient by layering 200 μl of each solution on top of each other from higher density (bottom) to lower density (top) in a centrifuge tube. Then, 200 μl of the P2 fraction was loaded on top of the layered gradient and the tubes were subjected to ultracentrifugation at 100,000 × *g* for 2 h. After ultracentrifugation, 100 μl of each fraction was removed by pipette carefully from top to bottom. The total fraction number was 16.

Ten microliters of each fraction was mixed with ATP (5 mM) and GTP (500 μM) in the physiological buffer (20 mM Tris-HCl, pH 7.5, 15 mM NaCl, 135 mM KCl, 5 mM Phosphate, 10 mM $MgCl_2$, 400 μM $ZnCl_2$ and 0.2 mg/ml BSA). The reaction mixture with a total volume of 60 μl was incubated at 37 ℃ for 2 hours, followed by heat inactivation at 95 ℃ for 5 min. After centrifugation at 20,000 × *g* for 5 min, supernatant was delivered into THP1-Lucia ISG cells to measure the cGAMP levels. The cGAMP produced inside Hela or THP1 cells during DNA transfection was theoretically fractionated into S20 (here we refer to as endogenous cGAMP), and the endogenous cGAMP production was measured and subtracted.

**Zinc Chelation in Cells**

L929 cells growing in six-well tissue culture plates were incubated with the zinc chelator TPEN at different concentrations for 2 hours before cells were transfected with HT-DNA by lipofectamine 2000 in DMEM medium (zinc free). Two hours after transfection, cells were lysed in 200 μl of hypotonic buffer (10 mM Tris-HCl, pH7.5, 5 mM KCl, and 3 mM $MgCl_2$) containing a protease inhibitor cocktail. cGAMP in the cell lysates was measured as described above.

**cGAS Thermo Shift Assay**

The recombinant hcGAS-FL protein was mixed with or without HT-DNA or $ZnCl_2$ (200 μM) in the Protein Thermal Shift Buffer (ThermoFisher) supplemented with a Protein Thermal Shift Dye (1000×) (ThermoFisher). Fluorescence reporter signals were recorded on a ViiA7 Real-Time PCR System (Applied Biosystems) with its Melt Curve option and ROX reporter type with Excitation Filter-Emission Filter x4 (580 ± 10)-m4 (623 ± 14) nm. Samples were subjected to a temperature gradient in the PCR machine from 25 ℃ to 99 ℃ with the Ramp rate of 0.05 ℃/s. The $T_m$ values were determined by fitting the melting curves to a Boltzmann sigmoidal equation using GraphPad Prism 7 software.

**Measurement of zinc ion binding to cGAS or DNA**

10 μM $ZnCl_2$ was incubated with various concentrations of DNA, cGAS, or both in a buffer containing 20 mM Tris-HCl, pH 7.5 and 150 mM NaCl. The mixture was passed through a 0.5 ml centrifugal filter with 30 kDa cutoff (Amicon), which was pre-rinsed with a buffer containing

20 mM Tris-HCl pH 7.5, 150 mM NaCl, and 10 μM $ZnCl_2$. After centrifuging the filters at 12,000 × *g* for 2 min, zinc ion concentration in the filtrate was measured by using a zinc quantification kit (Abcam). The data points were fitted to the equation of specific binding with Hill Slope in GraphPad Prism7. Kd is the cGAS concentration needed to achieve a half-maximum binding at equilibrium.

**Estimation of Cytoplasmic cGAS Concentration**

The cytoplasmic cGAS concentration of Hela cells was estimated by two methods as follows:

A. The proteins concentration of Hela cytoplasm is about 100 mg/ml (*28*). Based on the immunoblotting results of recombinant cGAS standards that were spiked into the lysates, endogenous cGAS in the cytoplasmic extract is approximately 0.1 ng per 20 μg cytoplasmic proteins. The cytoplasmic cGAS concentration would be: $\frac{100\ \mathrm{g/L} \times 0.1}{20000 \times 58814\ \mathrm{g/mol}} \approx 8.5 \times 10^{-9}\ \mathrm{M} =$ 8.5 nM.

B. Endogenous cGAS in the cytoplasmic extract is estimated to be approximately 0.1 ng per 20 μg cytoplasmic proteins. $10^5$ Hela cells contained about 20 μg cytoplasmic proteins. Thus, $10^5$ Hela cells contain about 0.1 ng cGAS. The cytoplasmic volume of each Hela cell is 1.4 $\times 10^{-12}$ L (*29*). The cytoplasmic cGAS concentration would be $\frac{0.1\ \mathrm{ng}}{10^5 \times 58814\ \mathrm{g/mol} \times 1.4 \times 10^{-12}\ L} \approx$ $12.1 \times 10^{-9}\ \mathrm{M} = 12.1$ nM.

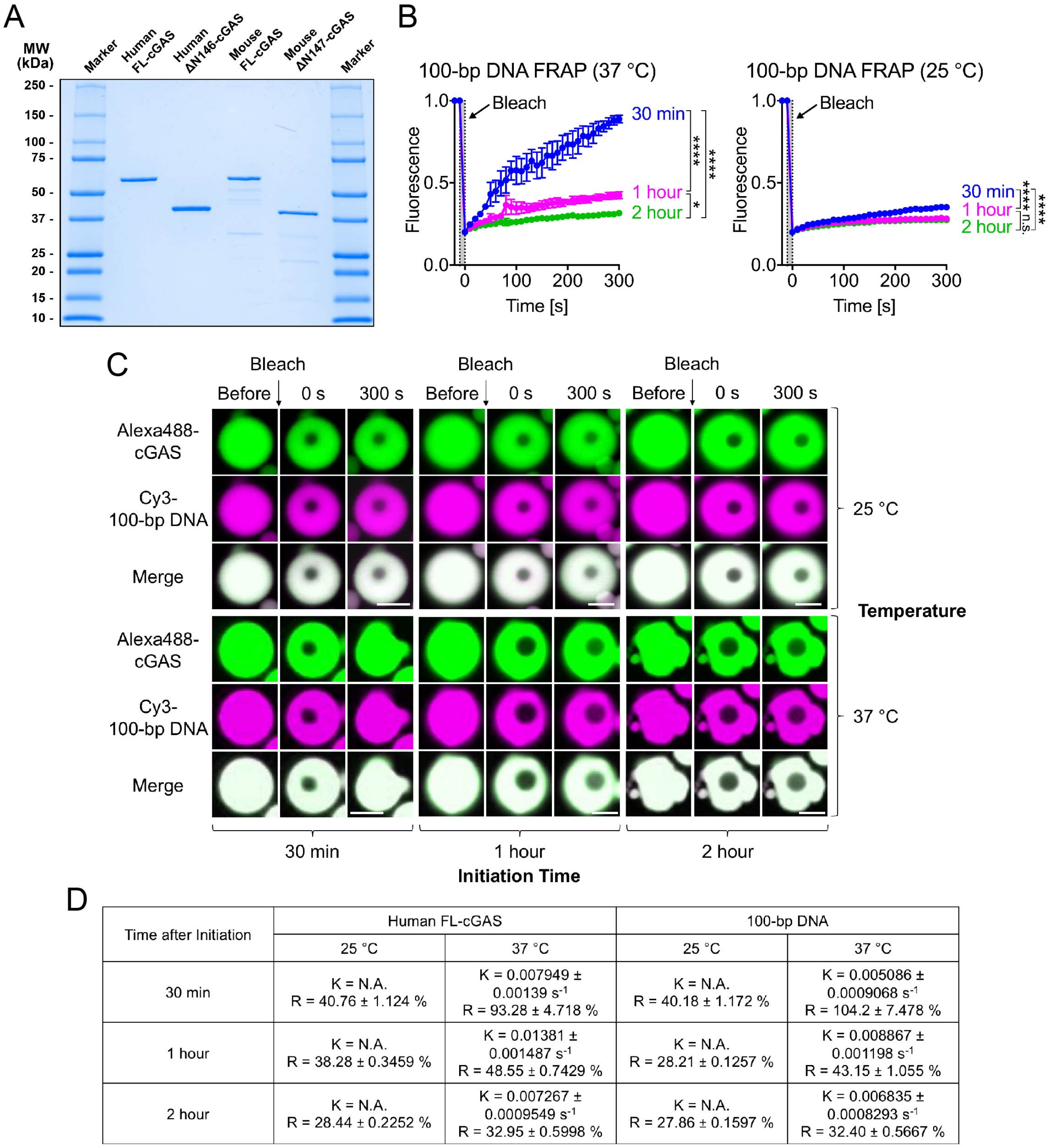


D

| Time after Initiation | Human FL-cGAS | | 100-bp DNA | |
|---|---|---|---|---|
| | 25 °C | 37 °C | 25 °C | 37 °C |
| 30 min | K = N.A.<br>R = 40.76 ± 1.124 % | K = 0.007949 ± 0.00139 $s^{-1}$<br>R = 93.28 ± 4.718 % | K = N.A.<br>R = 40.18 ± 1.172 % | K = 0.005086 ± 0.0009068 $s^{-1}$<br>R = 104.2 ± 7.478 % |
| 1 hour | K = N.A.<br>R = 38.28 ± 0.3459 % | K = 0.01381 ± 0.001487 $s^{-1}$<br>R = 48.55 ± 0.7429 % | K = N.A.<br>R = 28.21 ± 0.1257 % | K = 0.008867 ± 0.001198 $s^{-1}$<br>R = 43.15 ± 1.055 % |
| 2 hour | K = N.A.<br>R = 28.44 ± 0.2252 % | K = 0.007267 ± 0.0009549 $s^{-1}$<br>R = 32.95 ± 0.5998 % | K = N.A.<br>R = 27.86 ± 0.1597 % | K = 0.006835 ± 0.0008293 $s^{-1}$<br>R = 32.40 ± 0.5667 % |

**Figure S1. FRAP of cGAS–DNA liquid droplets formed in vitro.** (A) Coomassie blue staining of purified recombinant cGAS proteins. (B) FRAP of cGAS–DNA liquid droplets as measured by Cy3-DNA fluorescence intensity. Liquid droplets were formed by mixing Alexa Fluor 488-hcGAS (10 μM) and Cy3-DNA (100 bp, 10 μM) for 0.5 hr, 1 hr, or 2 hr, at which time a laser was used to photobleach the liquid droplets. Time 0 indicates the end of the photobleaching pulse

and the start of recovery. The recovery was allowed to occur at 25 °C (left) or 37 °C (right). Values are the mean ± SD. N = 3 cGAS–DNA liquid droplets. One-way ANOVA; p value: > 0.0332 (n.s.), 0.0332 (*), 0.0021 (**), 0.0002 (***), < 0.0001 (****). (C) Representative micrographs of FRAP experiments shown in Fig. 1F and Fig. S1B. Scale bar: 5 μm. (D) Statistical data of FRAP experiments in Fig. 1F and Fig. S1B. Data were fit to the single exponential model by GraphPad Prism 7 (see methods). K: exponential constant. R: normalized plateau after fluorescence recovery. Data are representative of at least three independent experiments.

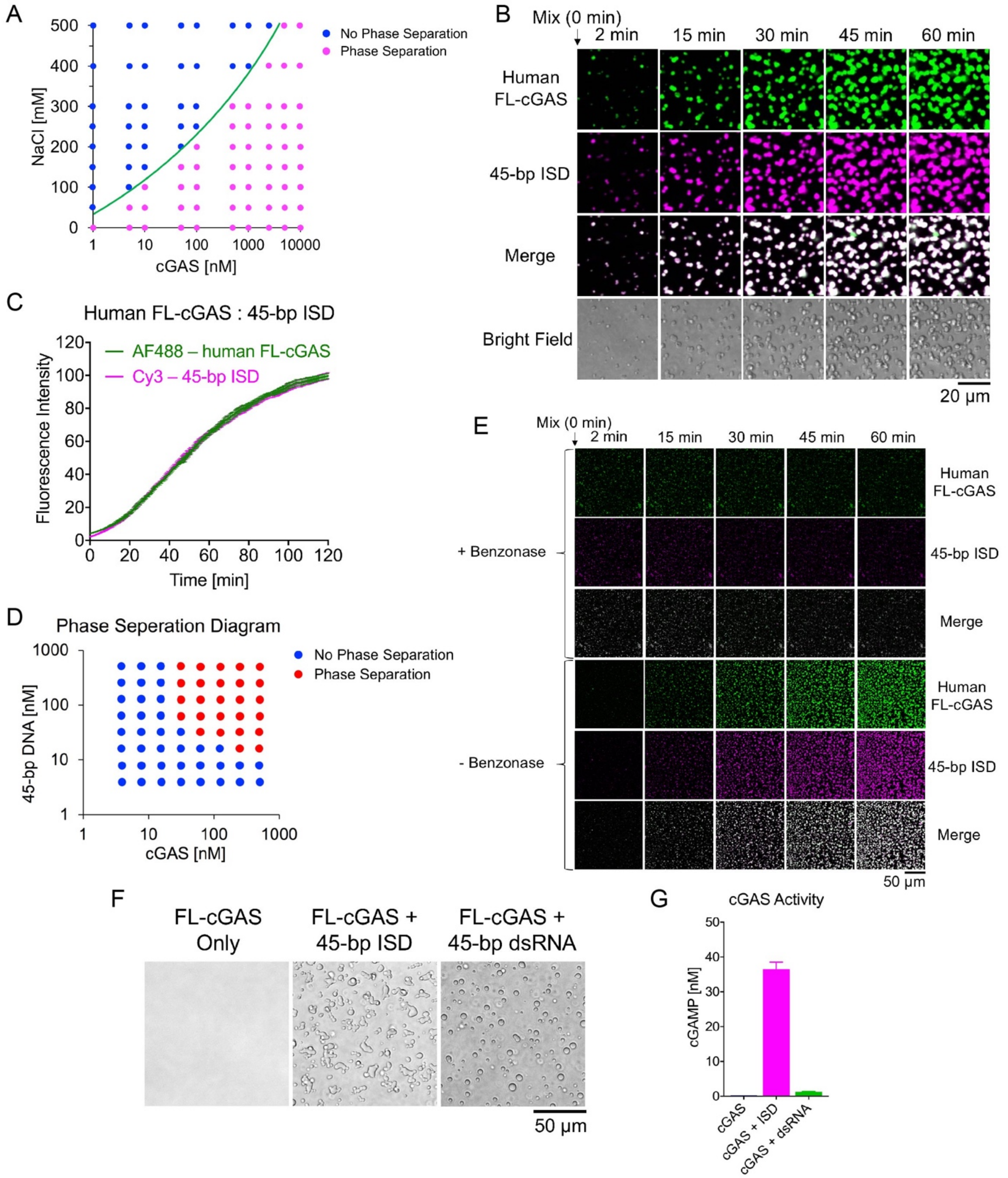


**Figure S2. DNA binding to cGAS induces the formation of liquid-like droplets.** (A) Phase separation diagram of hcGAS-FL and 100-bp DNA at indicated concentrations in the presence of different concentrations of NaCl. Blue dots: no phase separation; red dots: phase separation. (B) Time-lapse imaging of cGAS–DNA phase separation. Liquid droplets formed after mixing 10

μM full-length human cGAS (3% Alexa Fluor 488-labeled) with 10 μM 45 bp ISD (2% Cy3-labeled) and matured over the time course of 60 min. (C) Fluorescence intensities of cGAS–DNA liquid droplets that formed over the time course of 120 min. Data were normalized to 100% by the maximum fluorescence intensity at the plateau. Values shown are means ± SD. N = 4 images. AF488: Alexa Fluor 488. (D) Phase separation diagram of human cGAS-FL and 45 bp ISD at indicated concentrations. Blue dots: no phase separation; red dots: phase separation. (E) Time-lapse imaging of cGAS–DNA phase separation in the presence or absence of Benzonase (~15 nM). Human cGAS-FL: 5 μM; 45 bp ISD: 5 μM. (F) Bright-field images of human full-length cGAS (5 μM) phase separation with 45-bp ISD (5 μM) or 45-bp dsRNA (5 μM). (G) cGAMP production of human full-length cGAS with 45-bp ISD or 45-bp dsRNA. Error bars represent the variation range of duplicate assays. The images shown in (B), (E), and (F) are representative of all fields in the well. Data are representative of at least three independent experiments.

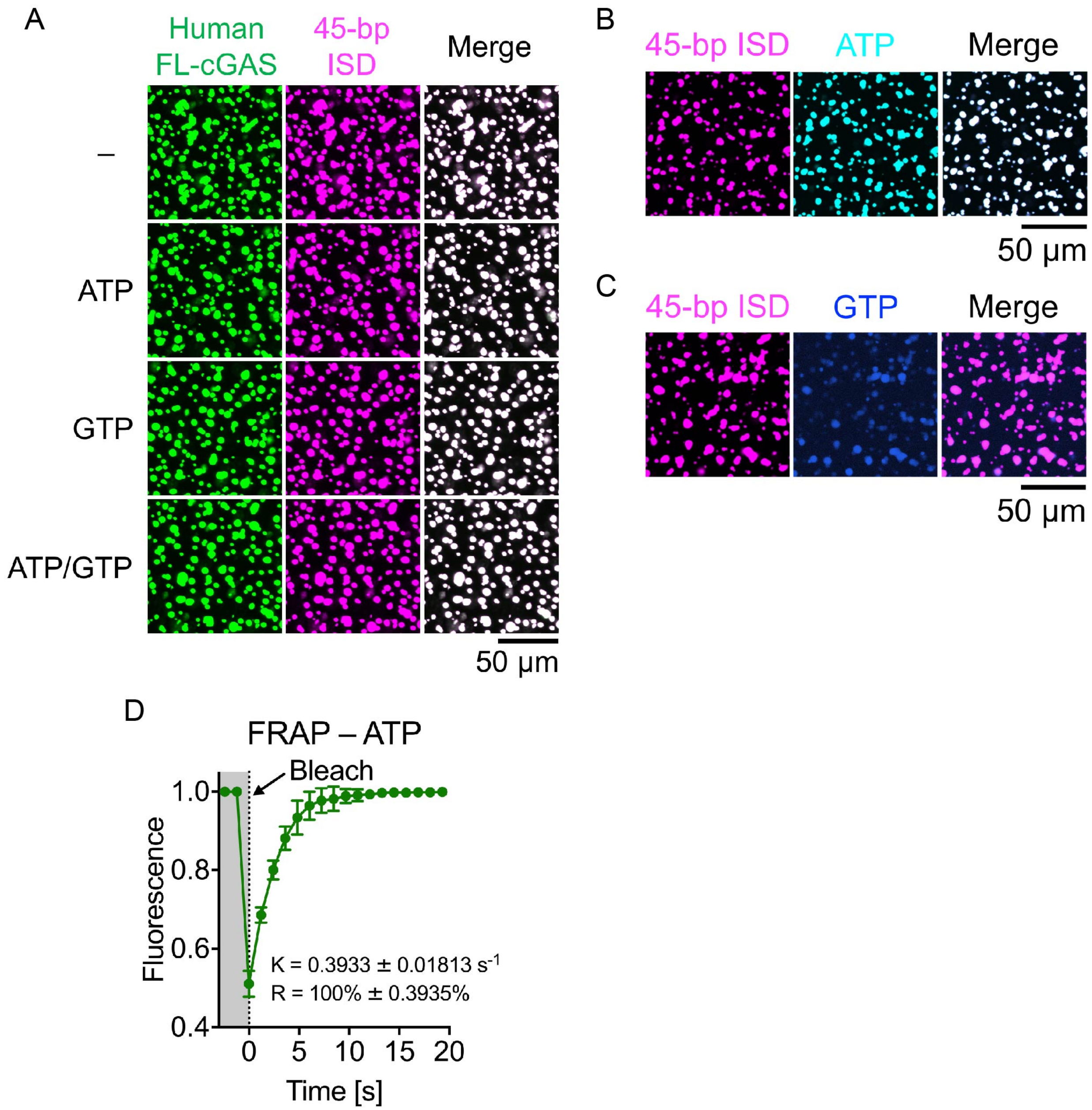


**Figure S3. ATP and GTP partition into the cGAS–DNA liquid droplets.** (A) cGAS–DNA phase separation is not affected by the presence of ATP (200 μM), GTP (200 μM), or both. Full-length human cGAS (20 μM; 3% Alexa Fluor 488-labeled) was incubated with 20 μM 45-bp ISD (2% Cy3-labeled) and images were taken after mixing for 60 min. (B) Imaging of ATP partitioning into cGAS–DNA condensates. Experiments were similar to (A), except that 250 nM Alexa Fluor 647-labeled ATP was added. (C) Imaging of GTP partitioning into cGAS–DNA condensates. Similar to (B) except that 2.5 μM N-Methylanthraniloyl (MANT)-GTP was used.

(D) FRAP of ATP in the cGAS–DNA condensates. Bleaching was performed 60 min after mixing cGAS (20 μM), ISD45 (20 μM), and 250 nM Alexa Fluor 647-labeled ATP. Time 0 indicates the end of photobleaching and the start of recovery. K: exponential constant. R: normalized plateau after fluorescence recovery. Shown are the mean ± SD. N = 3 liquid droplets. The images shown in (A), (B), and (C) are representative of all fields in the well. Data are representative of at least three independent experiments.

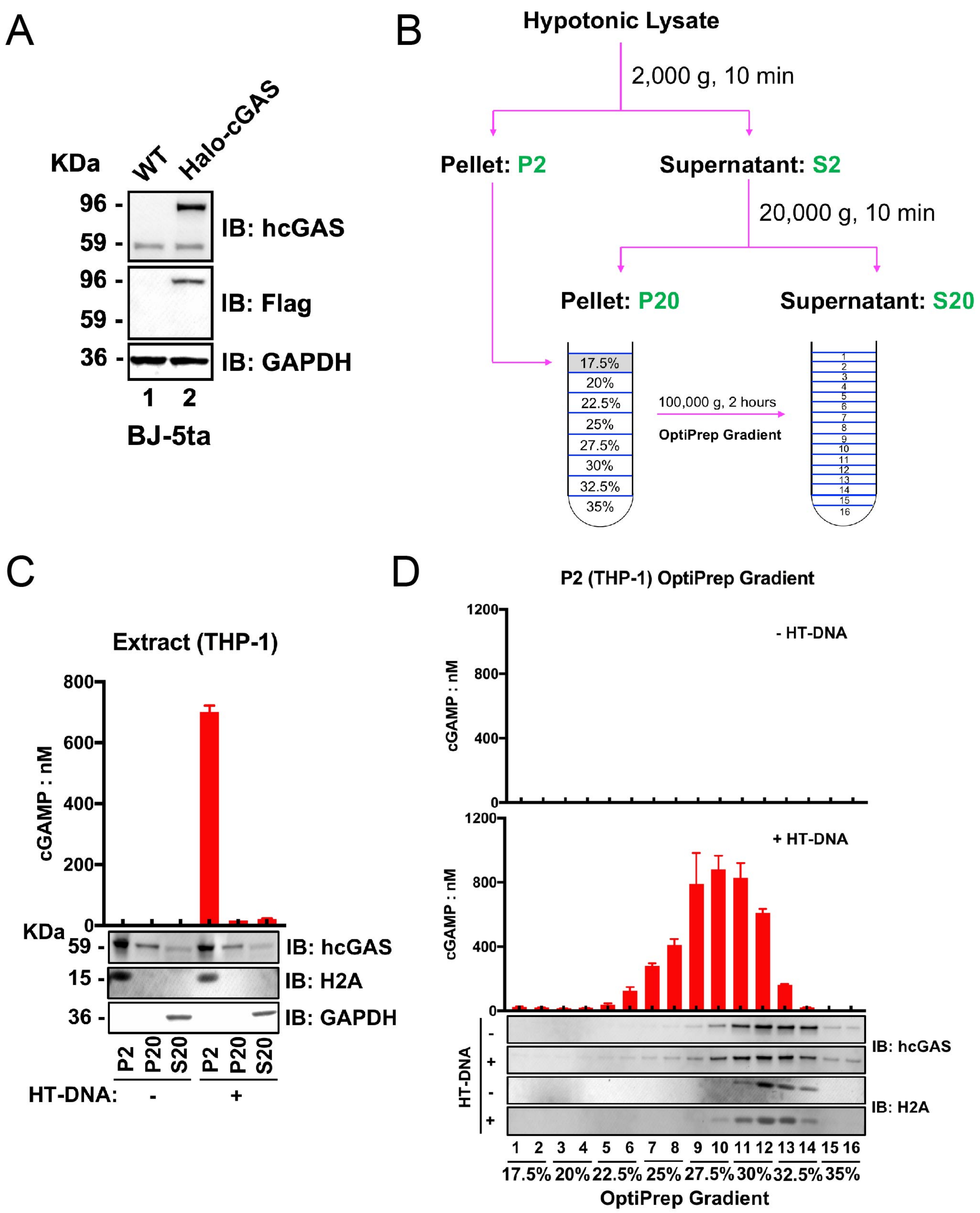


**Figure S4. DNA-induced phase separation and activation of cGAS in cells.** (A) Immunoblotting of lysates from BJ-5ta-Halo-cGAS cells. (B) Schematic of subcellular fractionation procedures. (C) Subcellular fractions of THP1 cells transfected with or without HT-

DNA were prepared by differential centrifugation as depicted in (B). Each fraction was incubated with ATP and GTP followed by cGAMP measurement. (D) THP1 P2 fractions from (C) were further separated by Optiprep gradient ultracentrifugation and cGAS activity in each fraction was measured as in (C). Fractions from cells not transfected with DNA had no cGAS activity. Error bars in (C) and (D) represent the variation range of duplicate assays. Data are representative of at least three independent experiments.

A

| | Human cGAS | | | Mouse cGAS | | | Chicken cGAS | | | Zebrafish cGAS | | | Xenopus | | |
|---|---|---|---|---|---|---|---|---|---|---|---|---|---|---|---|
| | FL | ΔN146 | N146 | FL | ΔN147 | N147 | FL | ΔN120 | N120 | FL | ΔN221 | N221 | FL | ΔN272 | N272 |
| pI | 9.54 | 9.18 | 10.91 | 9.61 | 8.94 | 11.66 | 9.54 | 9.05 | 11.84 | 9.25 | 8.80 | 9.61 | 9.55 | 9.03 | 10.08 |

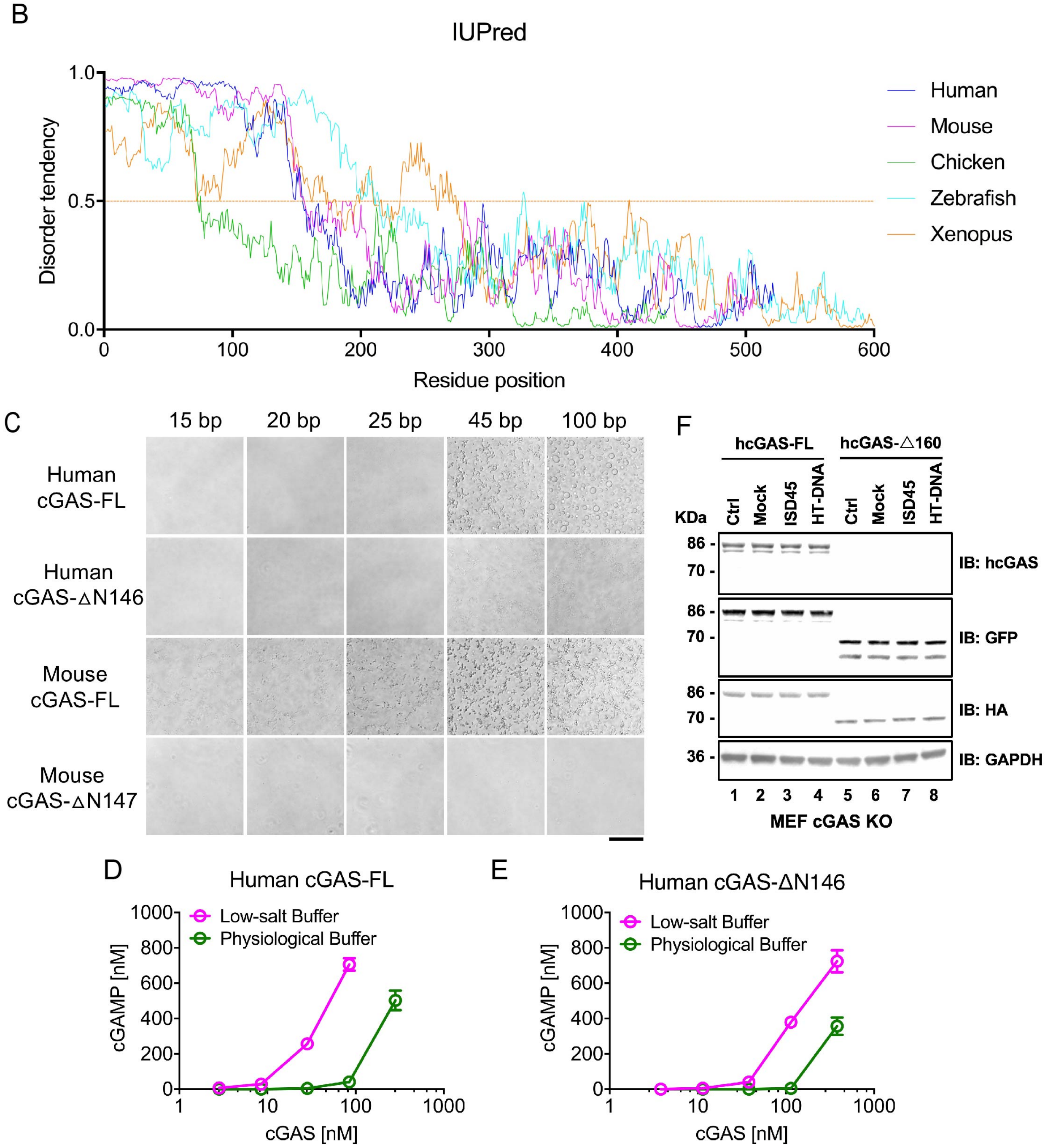


**Figure S5. The N-terminal DNA binding domain of cGAS promotes liquid phase separation and activation of cGAS.** (A) The N-terminus of cGAS is positively charged (high pI values; https://web.expasy.org/compute_pi/) (*30*). (B) The N-terminus of cGAS is intrinsically

unstructured (http://iupred.enzim.hu) (*30*). (C) Phase separation of indicated cGAS protein with DNA of different lengths in a buffer containing 300 mM NaCl. Scale bar: 100 μm. The images shown are representative of all fields in the well. (D & E) cGAMP production by different concentrations of recombinant full length (D) or ΔN human cGAS (E) in low-salt buffer or physiological buffer. These figures are different representations of Figure 3D and 3E. Shown are the mean ± SD. N = 3 assays. (F) Immunoblotting of cell lysates used in Fig. 3F. GFP-hcGAS-ΔN160 was detected by antibodies against GFP (fused to the N-terminus of cGAS) or HA (fused to the N-terminus of GFP), but not cGAS because the antibody recognizes an epitope at the N-terminus of cGAS. Data are representative of at least three independent experiments.

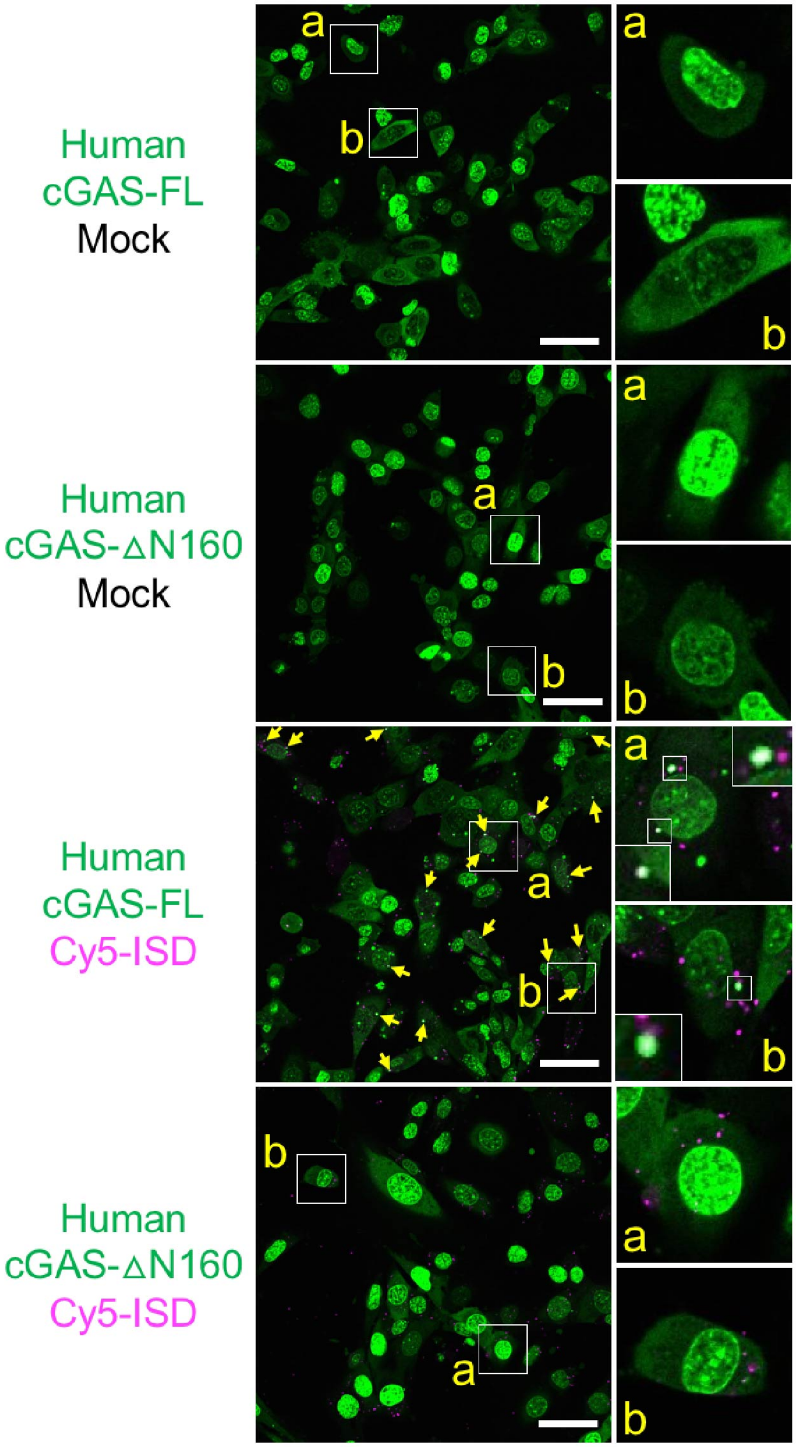


**Figure S6. The N-terminus of cGAS is important for its phase separation with DNA in cells.** Representative images of MEF cells expressing GFP-tagged full length human cGAS or ΔN160-cGAS after transfection of Cy5-ISD. Individual cells and cGAS–DNA puncta (Boxes) are enlarged. Scale bar: 50 μm. Quantification of the cGAS–DNA foci is shown in Figure 3F. These

images represent at least 5 fields examined. Data are representative of at least three independent experiments.

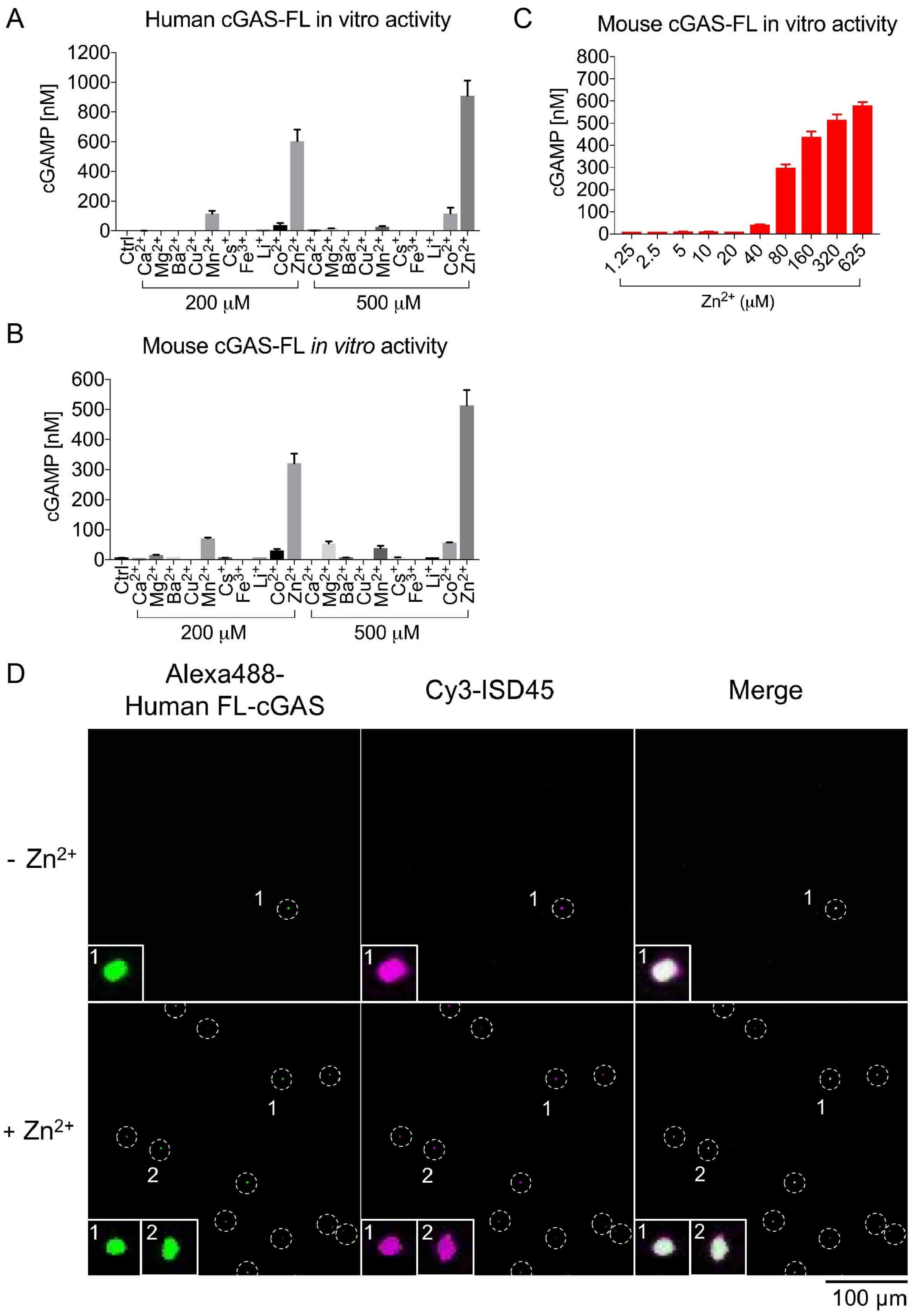
A
Human cGAS-FL in vitro activity
cGAMP [nM]
1200
1000
800
600
400
200
0
Ctrl
Ca2+
Mg2+
Ba2+
Cu2+
Mn2+
Cs+
Fe3+
Li+
Co2+
Zn2+
200 μM
500 μM
C
Mouse cGAS-FL in vitro activity
cGAMP [nM]
800
700
600
500
400
300
200
100
0
1.25
2.5
5
10
20
40
80
160
320
625
Zn2+ (μM)
B
Mouse cGAS-FL in vitro activity
cGAMP [nM]
600
500
400
300
200
100
0
200 μM
500 μM
D
Alexa488-
Human FL-cGAS
Cy3-ISD45
Merge
- Zn2+
+ Zn2+
100 μm

**Figure S7. Zinc ion promotes cGAS activity by inducing cGAS–DNA condensation.** (A) Human full-length cGAS (15 nM) was incubated with ATP (5 mM), GTP (300 μM) and HT-DNA (15 ng/μl) in physiological buffer supplemented with 200 μM or 500 μM of the indicated metal ions. cGAMP production was measured by a bioassay. (B) Similar to (A) except that mouse full-length cGAS (15 nM) was used. (C) Similar to (B) except that different concentrations of $Zn^{2+}$ were tested. Error bars in (A), (B), and (C) represent the variation range of duplicate assays. (D) Representative confocal images of cGAS–DNA condensates in the presence or absence of zinc (200 μM). The concentration of Alexa Fluor 488-labeled full-length human cGAS and 45-bp Cy3-labeled ISD was 20 nM each. Dashed circles highlight cGAS–DNA condensates. Insets showed the enlarged cGAS–DNA condensates. Quantification of the results are shown in Figure 4B. Data are representative of at least three independent experiments.

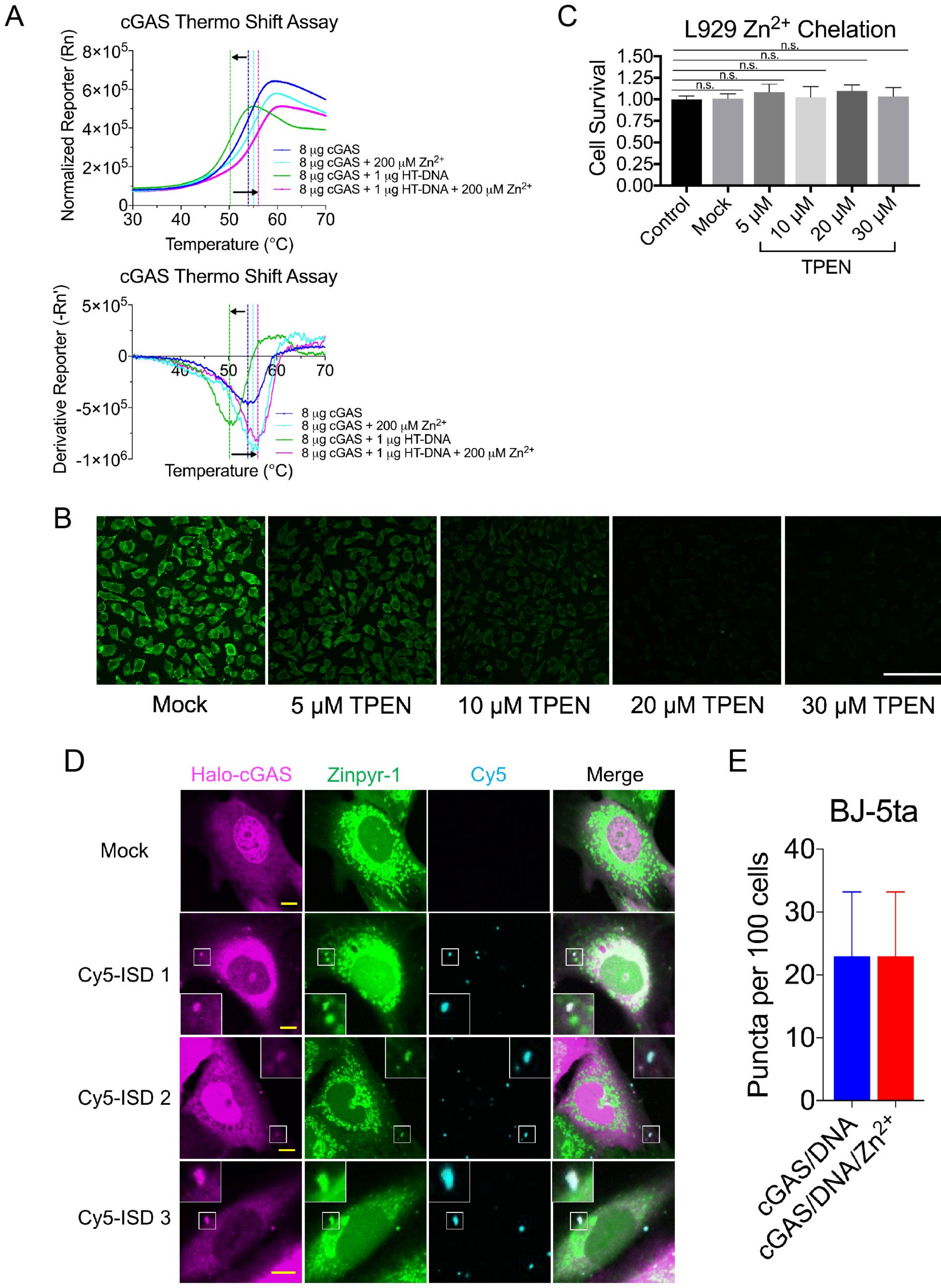
A
cGAS Thermo Shift Assay
Normalized Reporter (Rn)
8×10^5
6×10^5
4×10^5
2×10^5
0
30
40
50
60
70
Temperature (°C)
8 μg cGAS
8 μg cGAS + 200 μM Zn2+
8 μg cGAS + 1 μg HT-DNA
8 μg cGAS + 1 μg HT-DNA + 200 μM Zn2+
cGAS Thermo Shift Assay
Derivative Reporter (-Rn')
5×10^5
0
-5×10^5
-1×10^6
40
50
60
70
Temperature (°C)
8 μg cGAS
8 μg cGAS + 200 μM Zn2+
8 μg cGAS + 1 μg HT-DNA
8 μg cGAS + 1 μg HT-DNA + 200 μM Zn2+
C
L929 Zn2+ Chelation
Cell Survival
1.50
1.25
1.00
0.75
0.50
0.25
0.00
n.s.
n.s.
n.s.
n.s.
n.s.
Control
Mock
5 μM
10 μM
20 μM
30 μM
TPEN
B
Mock
5 μM TPEN
10 μM TPEN
20 μM TPEN
30 μM TPEN
D
Halo-cGAS
Zinpyr-1
Cy5
Merge
Mock
Cy5-ISD 1
Cy5-ISD 2
Cy5-ISD 3
E
BJ-5ta
Puncta per 100 cells
40
30
20
10
0
cGAS/DNA
cGAS/DNA/Zn2+

**Figure S8. Zinc ion promotes cGAS–DNA condensation.** (A) cGAS thermo shift assay to measure its stability in the presence of DNA, $Zn^{2+}$, or both. Upper panel: temperature-reporter signal plot. Lower panel: temperature-derivative reporter signal plot. (B) Zinpyr-1 (5 μM) was used to image free zinc ions in L929 cells treated with different concentrations of TPEN. Scale bar: 200 μm. The images shown are representative of all fields in the well. (C) Viability of L929 cells after 4 hours of zinc chelation by TPEN at the indicated concentrations. Values shown are means ± SD. N = 5. One-way ANOVA. (D) Cytoplasmic cGAS–DNA foci contain zinc. BJ-5ta cells stably expressing Halo–cGAS, which was labeled with TMR, were transfected with Cy5-ISD. The cells were treated with Zinpyr-1 followed by fluorescence microscopy. Representative images are shown, with the foci containing cGAS, DNA, and zinc highlighted (boxed). Scale bar: 10 μm. (E) Quantification of cGAS–DNA puncta and cGAS–DNA–$Zn^{2+}$ puncta as shown in (D). Values shown are means ± SD. N = 8 images. Data are representative of at least three independent experiments.

**Table S1. List of oligonucleotides used for cGAS condensation assay.**

| Name | Sequence (5□ to 3□) |
| --- | --- |
| 15-bp DNA | S: AAACAACACAACAAA |
| | AS: TTTGTTGTGTTGTTT |
| 20-bp DNA | S: AAAACAAACAACAAACAAAA |
| | AS: TTTTGTTTGTTGTTTGTTTT |
| 25-bp DNA | S: AAAACAAACAACACAACAAACAAAA |
| | AS: TTTTGTTTGTTGTGTTGTTTGTTTT |
| 45-bp DNA | S: AAACAAAAACAAAACAAACAACACAACAAACAAAACAAAAACAAA |
| | AS: TTTGTTTTTGTTTTGTTTGTTGTGTTGTTTGTTTTGTTTTTGTTT |
| 45-bp dsRNA | S: AAACAAAAACAAAACAAACAACACAACAAACAAAACAAAAACAAA |
| | AS: UUUGUUUUUGUUUUGUUUGUUGUGUUGUUUGUUUUGUUUUUGUUU |
| 45-bp ISD | S: TACAGATCTACTAGTGATCTATGACTGATCTGTACATGATCTACA |
| | AS: TGTAGATCATGTACAGATCAGTCATAGATCACTAGTAGATCTGTA |
| 45-bp Fluorescein-ISD | S: Fluorescein-<br>TACAGATCTACTAGTGATCTATGACTGATCTGTACATGATCTACA |
| | AS: TGTAGATCATGTACAGATCAGTCATAGATCACTAGTAGATCTGTA |
| 45-bp Cy3-ISD | S: Cy3-TACAGATCTACTAGTGATCTATGACTGATCTGTACATGATCTACA |
| | AS: TGTAGATCATGTACAGATCAGTCATAGATCACTAGTAGATCTGTA |
| 45-bp Cy5-ISD | S: Cy5-TACAGATCTACTAGTGATCTATGACTGATCTGTACATGATCTACA |
| | AS: TGTAGATCATGTACAGATCAGTCATAGATCACTAGTAGATCTGTA |
| 100-bp DNA | S: ACATCTAGTACATGTCTAGTCAGTATCTAGTGATTATCTAGACA<br>TACATCTAGTACATGTCTAGTCAGTATCTAGTGATTATCTAGACATGGACTCATCC |
| | AS: GGATGAGTCCATGTCTAGATAATCACTAGATACTGACTAGACATGTACTAGAT<br>GTATGTCTAGATAATCACTAGATACTGACTAGACATGTACTAGATGT |
| 100-bp Cy3-DNA | S: Cy3-ACATCTAGTACATGTCTAGTCAGTATCTAGTGATTATCTAGACA<br>TACATCTAGTACATGTCTAGTCAGTATCTAGTGATTATCTAGACATGGACTCATCC |
| | AS: GGATGAGTCCATGTCTAGATAATCACTAGATACTGACTAGACATGTACTAGAT<br>GTATGTCTAGATAATCACTAGATACTGACTAGACATGTACTAGATGT |

**Note:** S: sense strand; AS: anti-sense strand.

**Movie S1. Time-lapse imaging video of cGAS–DNA phase separation.** Liquid droplets formed after mixing 10 μM full-length human cGAS (3% Alexa Fluor 488-labeled) with 10 μM 100-bp DNA (2% Cy3-labeled) and matured over 120 min. Top left: Alexa Fluor 488 (full-length human cGAS); top right: Cy3 (100-bp DNA); bottom left: merge of Alexa Fluor 488 with Cy3; bottom right: bright field. Data are representative of at least three independent experiments.
